# Antiviral Activity of (1*S*,9a*R*)-1-[(1,2,3-Triazol-1-yl)methyl]octahydro-1*H*-quinolizines from the Alkaloid Lupinine

**DOI:** 10.3390/molecules29235742

**Published:** 2024-12-05

**Authors:** Zhangeldy S. Nurmaganbetov, Oralgazy A. Nurkenov, Andrei I. Khlebnikov, Serik D. Fazylov, Roza B. Seidakhmetova, Zhanar K. Tukhmetova, Altynaray T. Takibayeva, Gaukhar Khabdolda, Zhanar B. Rakhimberlinova, Aigul K. Kaldybayeva, Elvira E. Shults

**Affiliations:** 1 Laboratory of Synthesis of Biologically Active Substances, Institute of Organic Synthesis and Coal Chemistry, Karaganda 100008, Kazakhstan; nzhangeldy@yandex.ru (Z.S.N.); iosu8990@mail.ru (S.D.F.); 2 School of Pharmacy, Karaganda Medical University, Karaganda 100008, Kazakhstan; 3 Kizhner Research Center, Tomsk Polytechnic University, 634050 Tomsk, Russia; aikhl@chem.org.ru; 4 Department of Clinical Pharmacology and Evidence-Based Medicine, Karaganda Medical University, Karaganda 100008, Kazakhstan; rozabat@mail.ru; 5 Department of Biomedicine, Karaganda Medical University, Karaganda 100008, Kazakhstan; zhanar.tuhmetova@mail.ru (Z.K.T.); khabdoldag@mail.ru (G.K.); 6 Department of Chemistry and Chemical Technology, A. Saginov Karaganda Technical University, Karaganda 100027, Kazakhstan; altynarai81@mail.ru (A.T.T.); kargtu_tss@mail.ru (Z.B.R.); 7 Department of Pharmaceutical and Toxicological Chemistry, S.D. Asfendiyarov Kazakh National Medical University, Almaty 050012, Kazakhstan; aigul_240873@mail.ru; 8 N.N. Vorozhtsov Novosibirsk Institute of Organic Chemistry, Siberian Branch of the Russian Academy of Sciences, 630090 Novosibirsk, Russia

**Keywords:** alkaloid lupinine, (1*S*,9a*R*)-1-[(1,2,3-triazol-1-yl)methyl]octahydro-1*H*-quinolizines, antiviral activity, orthomyxoviruses, rimantadine, Tamiflu, molecular docking

## Abstract

Influenza is a disease of significant morbidity and mortality. The number of anti-influenza drugs is small; many of them stimulate the appearance of resistant strains. This article presents the results of assessing the antiviral activity of 1,2,3-triazole-containing derivatives of alkaloid lupinine for their ability to suppress the reproduction of orthomyxoviruses (influenza viruses: A/Vladivostok/2/09 (H1N1) and A/Almaty/8/98 (H3N2)). The ability of (1*S*,9a*R*)-1-[(1,2,3-triazol-1-yl)-methyl]octahydro-1*H*-quinolizines with aryl-, 4-((4-formylphenoxy)methyl)- or 4-((3-tert-butyl-5-ethyl-2-hydroxy-benzoyloxy)methyl)- substituents at the C-4 position of the triazole ring to reduce the infectivity of the virus when processing virus-containing material was established, indicating good prospects for the studied compounds as virucidal agents affecting extracellular virions. The experimental results demonstrated that the triazolyl lupinine derivatives exhibited varying degrees of affinity for both hemagglutinin and neuraminidase proteins. Furthermore, these compounds demonstrated inhibitory effects on the replication of influenza viruses with different antigenic subtypes. The obtained biological data are in agreement with the results of molecular docking, which showed strong binding energies of the investigated compounds under study with biological targets—hemagglutinin and neuraminidase proteins. Following the evaluation of antiviral efficacy among the studied triazolyl derivatives of lupinine, four compounds have been identified for subsequent comprehensive in vitro and in vivo investigations to further elucidate their antiviral properties.

## 1. Introduction

Three chemical classes of compounds against different viral targets are currently used as anti-influenza drugs. The adamantane derivatives rimantadine and amantadine have been used as first-choice antiviral drugs against community outbreaks of influenza A viruses for many years [1]. These compounds block the virally encoded protein M2, which acts as a proton channel required for the acidification of the virion core. The second class includes the neuraminidase inhibitors zanamivir (Relenza), oseltamivir (Tamiflu), peramivir (Rapiacta), and laninamivir (Inavir) [2]. They interfere with viral neuraminidase, which plays an essential role in the release of progeny virus particles from the surface of a host cell. The third class includes the nucleoside analogs ribavirin and favipiravir, which are antivirals with a broad range of activity that exhibit a suppressive effect against almost all RNA-genome human viruses [3]. However, ribavirin, a nucleoside analog, possesses numerous side effects, including the reduction of hemoglobin level, neutropenia, and so on [4]. Influenza virus is a dynamic pathogen with a rapid selection of drug-resistant strains. This fact underscores the serious challenge in the search and development of novel anti-influenza drugs with a broad range and alternative mechanism of activity.

The synthesis of chemical compound libraries followed by in vitro and/or in vivo screening of their biological activity is a traditional strategy to search for novel biologically active compounds. The use of natural sources for the development of new pharmacological agents has certain advantages, since often the activity of the natural precursor is already known, which is especially important for the creation of selective, “target-specific” drugs. The search for new biologically active compounds of plant origin for the treatment and prevention of immunosuppression, infectious diseases, and oncological and metabolic diseases remains an active part of pharmaceutical research [5]. In recent years, more than 40 new drugs of natural origin have appeared on the market, passed through all the way from research to clinical trials [3,4,5,6]. This attention of researchers to natural compounds is primarily due to the lower toxicity of plant-based preparations and a wider spectrum of action. Some medicinal compounds of plant origin (such as arglabine, taxol, and vinblastine) were found to effectively block the development of various viruses, including herpes simplex virus, influenza virus, human immunodeficiency virus, hepatitis C and B, and many others [4,5,6,7].

At the same time, in the treatment of infectious diseases, including viral ones, there is a centuries-old tradition of using medicinal plants, a significant part of which are alkaloid-bearing plants. 

**Figure 1:**
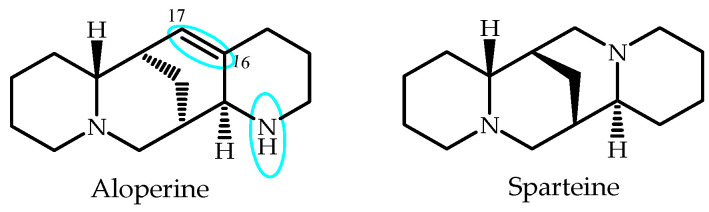
Quinolizidine-type antiviral alkaloids.

Quinolizidine alkaloids (lupinine, cytisine, lupanine, etc.) are of great interest due to their availability and biological activity. These alkaloids are present in many species of Fabaceae (*Lupinus*, *Genista*, *Cytisus*, *Sophora*, etc.) [8]. A targeted search for effective new therapeutic agents based on natural quinolizidine alkaloids, characterized by increased biological activity combined with low toxicity and less pronounced side effects, remains an urgent task. Modern pharmacological studies have shown that quinolizidine alkaloids isolated from *Sophora* plants, such as aloperine and sparteines (Figure 1), can inhibit influenza A virus infection (EC_50_ of 11.2–14.5 μM). The potency of quinolizidine alkaloids was improved by approximately 5-fold with chemical modifications (hydrogenation of the double bond) on the aloperine molecule [9]. It must be noted that several aloperine derivatives were identified as agents that inhibited the entry of SARS-CoV-2 (D614G variant) spike-protein-pseudotyped virus with an IC_50_ of 0.5 µM. The compound was also active against several other SARS-CoV-2 variants including Delta and Omicron [10,11]. 

A promising direction for creating new bioactive derivatives of the alkaloid lupinine is the synthesis of “hybrid molecules”, combining in their structure an alkaloid fragment and the 1,2,3-triazole pharmacophore. 1,2,3-Triazoles can be used as linker compounds, which are widely used in the synthesis of various biologically active substances. Derivatives of 1,2,3-triazole were used as antitumor [12,13,14,15,16,17], anti-inflammatory [18,19], antituberculosis [20,21,22,23], antimicrobial [24,25,26,27,28], and antiviral [29,30,31] agents, including the 1,2,3-triazolyl-substituted ribavirin analogs [30,31].

An analysis of the literature data on the chemical transformations of lupinine showed that its various ester, amine, imide, halogen, thio-, and *O*-acyl derivatives were previously synthesized and evaluated for their biological activity [32,33,34,35,36]. Recently, based on the transformation of (-)-lupinine, we developed a preparative synthesis of (1*S*,9a*R*)-1-[(1,2,3-triazol-1-yl)-methyl]octahydro-1*H*-quinolizines with various substituents at the C-4 position of the 1,2,3-triazole ring [37,38,39,40]. 

In this work, we present a pharmacological investigation of (1*S*,9a*R*)-1-[(1,2,3-triazol-1-yl)methyl]octahydro-1*H*-quinolizines with aryl-, 4-((4-formylphenoxy)methyl)-, or 4-((3-*tert*-butyl-5-ethyl-2-hydroxy-benzoyloxy)methyl)- substituents at the C-4 position of the triazole ring derived from the alkaloid lupinine for their ability to suppress the replication of orthomyxoviruses (influenza viruses: A/Vladivostok/2/09 (H1N1) and A/Almaty/8/98 (H3N2)) to expand the arsenal of antiviral agents. To confirm the obtained biological results, molecular docking of the studied compounds was carried out based on AutoDock Vina 1.2.1software with biotargets of antiviral activity—hemagglutinin and neuraminidase protein models. 

## 2. Results and Discussion

**Scheme 1:**
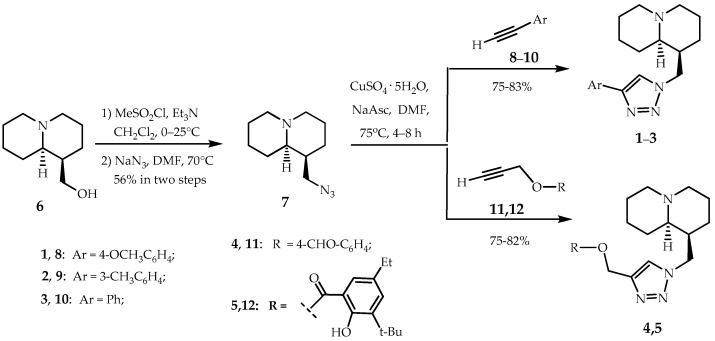
Synthesis of (1*S*,9a*R*)-1-[(1,2,3-triazol-1-yl)methyl]octahydro-1*H*-quinolizines **1**–**5** from (-)-lupinine **6**.

The synthesis of lupinine derivatives **1**–**5** was carried out from the alkaloid (-)-lupinine 6 by the reaction of lupinine azide **7** [37] with aryl alkynes **8**–**10**, 4-(prop-2-ynyloxy)benzaldehyde **11** or prop-2-ynyl 3-*tert*-butyl-5-ethyl-2-hydroxybenzoate **12** under the conditions of Cu(I)-catalyzed 1,3-dipolar cycloaddition (Scheme 1). 

The structure of the compounds **1**–**5** was fully confirmed by ^1^H and ^13^C NMR spectroscopy, mass spectrometry, and X-ray structural analysis for compounds **1** and **2** [37]. 

### 2.1. Investigation of Antiviral Activity

#### 2.1.1. Cytotoxicity and Chicken Embryo Lethality of the Tested Compounds

The acute toxicity of the studied compounds in various doses was assessed using different in vitro models (erythrocytes; chicken embryos). The dose range was determined primarily by the interval of acceptable values for the number of samples used in further studies of the antiviral activity. The analysis of the acute cytotoxicity of lupinine derivatives **1**–**5** in vitro was carried out in the concentration range of 0.03–1% (from 0.03 mg to 1 mg in 100 μL), which corresponded to typical doses of other alkaloid compounds investigated for antiviral properties.

The cytotoxicity of the samples was determined by studying the effect of various doses of the compounds on cell viability using the method of detecting dehydrogenase activity (MTT assay). It was established that in the studied dose range, the LD_50_ value was not achieved in any of the studied samples. 

The analysis of the acute toxicity of the studied compounds on a model of 10-day chicken embryos was carried out in the dose range of 0.003–0.4 mg/chicken embryo (0.06–8 mg/kg). It was established that at the maximum dose (0.4 mg/chicken embryo), a noticeable toxicity was not achieved. Therefore, further study of the antiviral activity was carried out in doses of 0.4 mg/chicken embryo or lower.

Thus, when determining acute toxicity in vitro, on a model of 10-day chicken embryos, the investigated compounds did not reveal toxic properties at the maximum dose studied.

#### 2.1.2. Hemagglutinating Activity 

To determine the effect of compounds **1**–**5** on hemagglutinating activity, the maximum dose used was 1 mg/100 μL.

**Figure 2:**
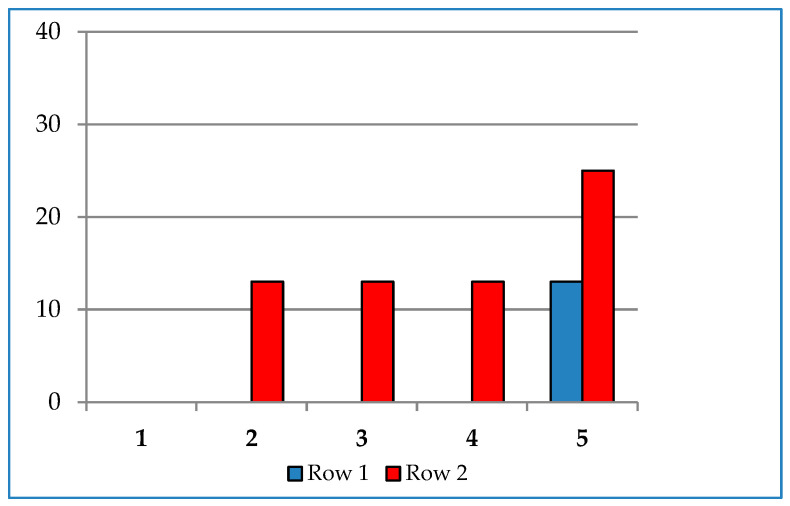
Influence of the studied compounds on hemagglutinating activity. 1st row—A/Almaty/8/98 (H3N2); 2nd row—A/Vladivostok/2/09 (H1N1). The *y*-axis is the percentage of activity suppression.

It was established (Figure 2) that none of the studied samples was capable of inhibiting the hemagglutinating activity of the influenza virus by more than 50%. 

However, {1-[((1*S*,9a*R*)-octahydro-2*H*-quinolizin-1-yl)methyl]-1*H*-1,2,3-triazol-4-yl}methyl-3-*tert*-butyl-2-hydroxy-5-ethylbenzoate (**5**) was capable of suppressing the hemagglutinating activity of the influenza A/Vladivostok/2/09 (H1N1) virus by no less than 25%.

#### 2.1.3. Neuraminidase Activity

**Figure 3:**
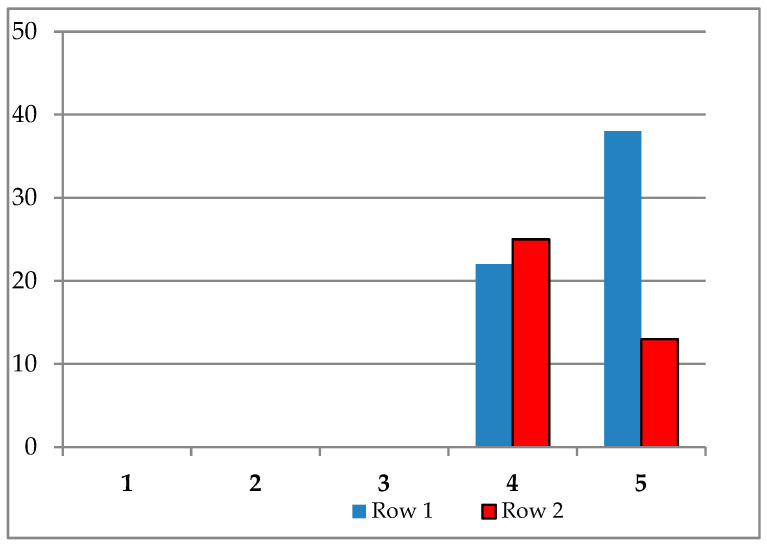
The influence of the studied samples on neuraminidase activity. 1st row—A/Almaty/8/98 (H3N2); 2nd row—A/Vladivostok/2/09 (H1N1). The *y*-axis is the percentage of activity suppression.

To determine the effect of lupinine derivatives **1**–**5** on neuraminidase activity, the maximum dose of 1 mg/100 μL was used. It was established (Figure 3) that compounds **4** and **5** were able to alter the neuraminidase activity of influenza viruses. It was shown that these two triazole-containing lupinines suppressed neuraminidase activity by no more than 40%. 

#### 2.1.4. Virucidal Activity

The virucidal activity is one of the main approaches to determining the effectiveness of drugs with antiviral activity. The activity of compounds **1**–**5** was determined at the dose of 0.4 mg/chicken embryo. The following influenza virus strains were used as model viruses: A/Almaty/8/98 (H3N2); A/Vladivostok/2/09 (H1N1).

It was established that the virucidal activity of the lupinine derivatives **1**–**5** varied from 0.0 to 2.0 lg (Table 1). 

In our experiments, compounds **1**, **4**, and **5** were able to reduce the infectivity of the influenza virus by more than 1 lg, which means a loss of 90% of the virus infectivity. The obtained results show the potential of these derivatives as virucidal agents affecting extracellular virions.

The substituent in the C-4 position of the 1,2,3-triazole ring has an influence on virucidal activity. The introduction of a 4-phenyl substituent in the triazole ring (compound **3**) is detrimental to this activity. The presence of an activating methoxyl group in the 4 position of the phenyl ring (compound **1**) induces a beneficial effect to the activity, while an activating methyl group in position 3 twice decreased the virus infectivity titer (compound **2**). This confirmed that position 4 of the phenyl ring (compound **1**) confers a better potency than position 3 (compound **2**). In must be noted that the introduction of an aryl substituent through the O-methyl (ester) group in the C-4 position of the triazole ring led to the more active compound **4**. Nevertheless, the aroyloxymethyl substituent in the C-4 position of the triazole ring (compound **5**) was generally more active in reducing of the infectivity of the influenza virus than the ester one (compound **4**). 

#### 2.1.5. Comparative Study of the Antiviral Activity of the Lupinine Derivatives

To determine the virus inhibitory activity, influenza virus strains with different antigenic formulae were taken: A/Almaty/8/98 (H3N2); A/Vladivostok/2/09 (H1N1).

Rimantadine^®^ (Olainfarm, Olaine, Latvia), alpha-methyltricyclo[3.3.1.1/.7]decan-1-methanamine (in the form of hydrochloride)) was used as a comparison drug at a dose of 10 µg /mL and Tamiflu^®^ (Oseltamivir, Hoffmann-La Roche, Basel, Switzerland, (3*R*,4*R,*5*S*)-4-acetylamino-5-amino-3-(1-ethylpropoxy)-1-cyclohexene-1-carboxylic acid ethyl ether, phosphate)) at a dose of 6 µg /mL.

To determine the chemical therapeutic index (CTI), the virus inhibitory activity of samples was studied at concentrations from 0.0016% to 0.2%, which corresponded to doses of 0.003–0.4 mg per chicken embryo (0.06–8 mg/kg) (Table 2).

It was found that all the studied compounds exhibited varying degrees of suppressing the replication of influenza viruses with different antigenic structures. However, when compared with the activity of the commercial drugs Tamiflu and Rimantadine, it was shown that four of the studied triazole-containing lupinines (compounds **1**, **2**, **4**, and **5**) are promising for further research, as they are superior in activity to commercial drugs, regardless of the antigenic structure of influenza and its sensitivity to antiviral drugs. Due to the small number of compounds, it is difficult to draw definite conclusions on structure–activity relationships. However, it can be noted that the antiviral activity strongly depends on the nature of the substituent in the C-4 position in the triazole ring. The presence of a methyl or methoxyl group in the 4-aryltriazole substituent, no matter where it was attached to the phenyl ring, induced a beneficial effect to the activity (compounds **1** and **2**). The methyloxy linker chain in the triazole C-4 position also conferred better potency than the 4-phenyltriazolyl substituent (compound **4**). On the other hand, the presence of a benzoyloxy methyl (more polar) substituent in the 4 position of the 1,2,3-triazole ring resulted in the increase in activity among all derivatives (compound **5**). Similar effects of the substituent in the 4 position of the triazole ring were observed in neuraminidase inhibition assays for 5′-substituted 1,2,3-triazolylated oseltamivir [41] and zanamivir [42] derivatives. Compounds with the methoxy linker chain and a bulky substituent in the 4 position of the triazole ring generally had a higher activity compared with the 4-phenyl series.

#### 2.1.6. Molecular Docking

We performed a docking study of compounds **1**–**5** using the structures of key viral proteins: hemagglutinin (HA) and neuraminidase (NA). While HA is a major surface glycoprotein of influenza viruses that plays a crucial role in viral entry and infection [43], NA functions as a sialidase, cleaving sialic acid residues from host cell receptors and newly formed viral particles [44]. This enzymatic activity is essential for preventing viral aggregation and facilitating the release of progeny virions from infected cells [45]. We used the structures 1RUY (1930 swine H1 hemagglutinin [46]) and 3BEQ (neuraminidase of 1918 influenza virus H1N1 strain [47]) from the Protein Data Bank as biotargets for docking. Both HA and NA have several targeting areas [48,49,50]. Therefore, a docking variant with a systematic search for potential binding sites was used based on the application of the AutoLigand methodology [51].

**Figure 4:**
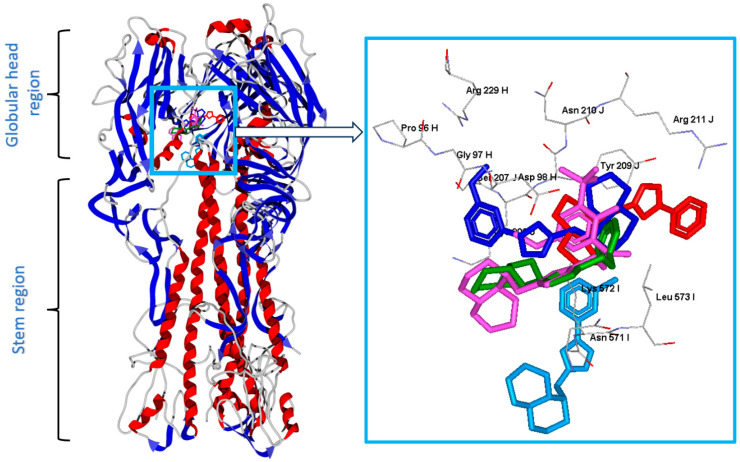
The general backbone view of swine H1 hemagglutinin (PDB: 1RUY) with the docking poses of compounds **1**–**5** in a cavity near the vestigial esterase and receptor binding subdomains in the globular head region (Site 1) (**left**). The large-scale view of the superimposed docking poses with amino acid residues within 4 Å from the pose of compound **1** (**right**). The docked molecules are colored in blue **1**, light blue **2**, red **3**, green **4**, and magenta **5**.

Our docking results for the 1RUY hemagglutinin structure show that ligands **1**–**5** bind well within a cavity (Site 1) located in the vicinity of the vestigial esterase and receptor binding subdomains [41] in the globular head region of HA (Figure 4). The binding energies of the investigated compounds to Site 1 evaluated using AutoDock Vina 1.2.1 software range from −6.7 to −7.7 kcal/mol. The energies are summarized in Table 3, which also lists the formed hydrogen bonds and the amino acid residues within 3 Å of each ligand pose.

Molecules **1**–**5** within Site 1 are surrounded by amino acid residues of three protein chains (H, I, J) present in the 1RUY structure. The compounds under study interact with most of these residues via van der Waals forces. However, compounds **2**–**5** are also anchored in Site 1 via hydrogen bonding (Table 3).

**Figure 5:**
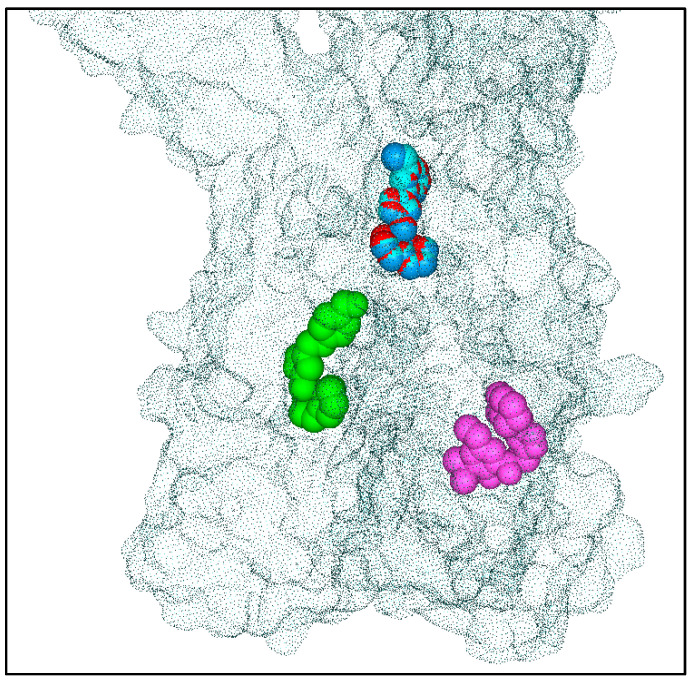
The stem region of hemagglutinin (PDB: 1RUY) shown as the dotted surface with the docking poses of compounds **1** (blue), **2** (light blue), **3** (red), **4** (green), and **5** (magenta). The poses of molecules **1**–**3** are overlaid within Site 2, while compounds **4** and **5** occupy Sites 3 and 4, respectively.

It should be noted that the studied lupinine derivatives, according to the docking results, bind most tightly to sites within the stem region of HA (Figure 5) (Sites 2–4 found using the AutoLigand procedure), having binding energies from −8.2 (compound **3**) to −9.2 kcal/mol (compound **5**). The main characteristics of these poses, including the closest amino acid residues of HA, are given in Table S1. The stem region is responsible for membrane fusion [52]. Hence, the binding with Sites 2–4 can be also very important for the antiviral activity of compounds **1**–**5**.

**Figure 6:**
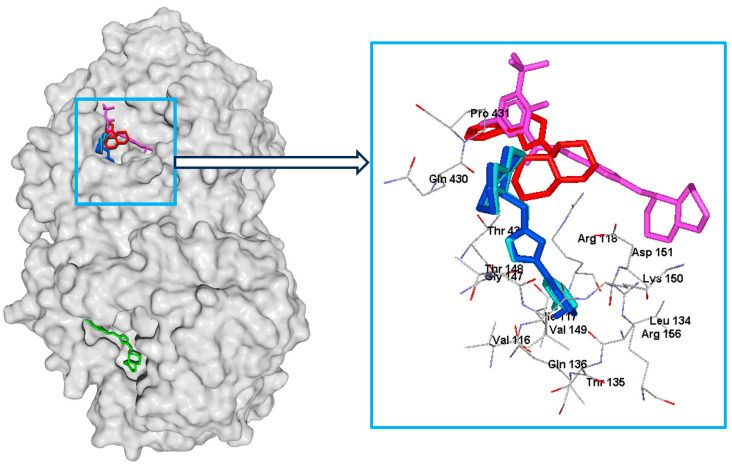
The general surface view of neuraminidase of 1918 influenza virus H1N1 with the docking poses of compounds **1**–**5** in the substrate-binding sites in chains A and B (PDB: 3BEQ) (**left**). The large-scale view of the superimposed docking poses in the site of chain B with amino acid residues within 4 Å from the pose of compound **1** (**right**). The docked molecules are colored in blue **1**, light blue **2**, red **3**, green **4**, and magenta **5**.

The application of the AutoLigand methodology to neuraminidase of the 1918 influenza virus H1N1 led to the docking poses within the substrate-binding site described by Xu and co-authors [47] located in chain B (compounds **1**–**3**, **5**) or in the counterpart of this site in chain A of the tetrameric PDB structure 3BEQ (Figure 6). The characteristics of the docking poses are presented in Table 3.

The binding energies evaluated with AutoDock Vina software are between −7.5 (compound **3**) and −9.6 kcal/mol (compound **5**). These results show that most of the studied compounds have potential for use as neuraminidase inhibitors. Notably, the weakest binding to both biotargets (HA and NA) was obtained for compound **3**, which is consistent with the results of our antiviral activity study.

## 3. Materials and Methods

In the present work, we used five derivatives of lupinine **1**–**5** (Scheme 1, Table 4). The synthesis is described in our previous works [37].

### 3.1. In Vitro Antivirus Assays

#### 3.1.1. The Study of Toxicity

The study of the toxicity of lupinines **1**–**5** was carried out in 3 variants:

The toxicity of compounds **1**–**5** was studied when treated with 2% rooster erythrocytes, as well as on a primary culture of chicken embryo fibroblasts and on 10-day developing chicken embryos. The studied samples were diluted in a minimal volume of DMSO; after dissolution, a series of 2-fold dilutions were prepared in phosphate buffer, pH 7.2. The preparation of the studied doses of the compounds began with determining their solubility limit. It was shown that the maximum amount of the samples soluble in alcohol or DMSO was 100 mg/mL, so doses not exceeding these values were used for further experiments. Serial dilutions of samples (10% stock solution in alcohol) were conducted in buffer pH 7.2.

The primary determination of the toxic (hemolytic) dose of the studied lupinines was carried out using 2% rooster erythrocytes. For this purpose, the studied samples were mixed in a ratio of 1:5 with a 2% solution of rooster erythrocytes. After 120 min of incubation at 37 °C, an equal volume of cold physiological solution was added and centrifuged for 5 min at 13,000 rpm. The optical density of the supernatant liquid was measured on an M200 spectrophotometer (Tecan, Switzerland) at 412 nm. Based on the data obtained, we calculated the toxic dose of the drug (TK_50_), at which 50% lysis of red blood cells occurs. Based on the TK_50_ value, the working concentrations of the drug were calculated.

The effect of the studied samples, in different doses, on cell viability (primary culture of chicken embryo fibroblasts, 104 cells/well) was determined by the detection of dehydrogenase activity (MTT assay).

The MTT assay is based on the ability of living cell dehydrogenases to reduce the uncolored forms of 3-(4,5-dimethylthiazol)-2-yl-2,5-diphenylterazole (MTT reagent) to blue crystalline pharmazan, soluble in dimethyl sulfoxide.

MTT solution (Calbiochem, Burlington, MA, USA) was prepared in physiological solution at a concentration of 0.5 μg/mL. The MTT solution was added to the wells containing cells, previously washed from the medium, to a volume of 0.1 mL. After 1 h of contact of MTT with cells, the wells were washed and filled with 0.1 mL of DMSO, after which the optical density in the wells was measured on an M200 spectrophotometer (Tecan, Switzerland) at a wavelength of 535 nm. Based on the data obtained, the toxic dose of the samples (TC_50_), at which 50% cell destruction occurs, was calculated. Based on the TK_50_ value, the working concentrations of the samples were calculated.

The toxicity of the studied samples in different doses to 10-day-old chicken embryos (embryotoxicity) was determined by inoculating 0.2 mL of the studied samples into the chorion-allantoic cavity of chicken embryos. Toxicity of samples was detected by the death of chicken embryos within 4 days after inoculation of materials.

#### 3.1.2. Study of the Virus-Inhibiting Activity of the Samples Against Influenza Viruses (H1N1, H3N2 Strains) on a Chicken Embryo Model

Assessment of the ability of the studied compounds to suppress the infectivity of the influenza virus (on a model of 2 strains).

For the determination of virucidal activity, samples in different doses were mixed with an equal volume of influenza virus with an infectivity titer of at least 10^−7^ EID_50_/0.2 mL. The mixture was incubated for 30 min at 37 °C. Several serial 10-fold dilutions were prepared, and starting with the maximum dilution, 10-day-old chicken embryos (at least 4 chicken embryos) were infected with 0.2 mL. After 24–48 h, data on the presence of the virus were determined by hemagglutination in the allantoic fluid of the embryo.

Cumulative data (cumulative indicator) were determined. When calculating the cumulative data for the number of healthy embryos, the higher number was added to the lower one, starting with the smallest. It was believed that if the embryo did not show the presence of the virus at a higher dose, then it would not show up at a lower dose. When calculating the total data on the number of dead embryos, the lower figure was added to the higher one. It was believed that if the embryo was infected with a lower dose, then it would become infected with a higher dose.

The percentage of infection efficiency was determined as follows: total number of infected ≈ 100/(total number of infected + total number of healthy).

The coefficient of proportionality (CP) was determined:

$$CP=\frac{\left(\;infection\;percentage\;with\;HCD-50\right)\;\;\;\;\;\;\;\;\;\;\;\;\;\;\;\;\;\;\;\;\;\;\;\;\;\;\;\;\;\;\;\;\;\;\;\;\;\;\;\;\;\;\;\;\;\;\;\;\;\;\;\;\;\left(54-50\right)}{\left(\;infection\;percentage\;with\;HCD-infection\;percentage\;with\;LCD\right)\;\;\;\;\;\;\;\left(54-29\right)}=0.16$$

10^−8^—highest critical dose (HCD), 54%
10^−9^—lowest critical dose (LCD), 29%
T = HCD – CP = 10^−8–0.16 ^ = 10^−8.16^ = EID_50_/0.2 mL.
8—shows how many characters there will be in the final result (+1).
16—mantissa. Using the mantissa, in accordance with the antilogarithm function, we find the number (1445) 1: 1:144,500,000 EID_50_/0.2 mL or 1 EID_50_ = 1:144,500,000 in 0.2 mL.

If the infectivity titer is reduced by more than 1 lg, then the substance is considered capable of suppressing 99% of the virus in the sample.

#### 3.1.3. Evaluation of the Ability to Suppress the Infectivity of the Influenza Virus (on a Model of 2 Strains)

The hemagglutinating activity of viruses was determined according to the standard method [53] using a 0.75% suspension of chicken erythrocytes. When determining the effect of the studied sample on hemagglutinating properties, the sample in different doses was mixed with the virus, and after 30 min of incubation at 37 °C, the hemagglutinating activity was determined using the standard method.

The determination of the neuraminidase activity of the studied samples was carried out using a fluorescent kit in accordance with the manufacturer’s recommendations (AbCam, Cambridge, UK). The non-fluorescent neuraminidase substrate becomes highly fluorescent when cleaved by neuraminidase. The signal is easily read by fluorescence at Ex/Em = ~320/~450 nm.

### 3.2. Docking Computations

The 3D molecular models of compounds **1**–**5** (ligands) were prepared using ChemOffice 16 software. The protein structures of 1930 swine H1 hemagglutinin and neuraminidase of 1918 H1N1 strain were downloaded from the Protein Data Bank (PDB). The PDB codes of the structures were 1RUY and 3BEQ, respectively. The ligand and protein structures were imported into the AMDock suite of programs (Version 1.5.2) [54] and converted to PDBQT format. The protonation states of the ligands and the proteins were adjusted to pH 7.4 using the PROPKA algorithm [55] implemented in AMDock. Locations of the potential binding sites in the protein structures were found with the use of the AutoLigand method [51]. Docking of compounds **1**–**5** into the binding sites was performed with the default options of the AutoDock Vina module embedded in AMDock. The collected docking poses were analyzed and visualized using Molegro 6.0 software.

## 4. Conclusions

The developed 4-substituted (1*S*,9a*R*)-1-[(1,2,3-triazol-1-yl)methyl]octahydro-1*H*-quinolizines exhibited influenza virus inhibitory activity, and the ability to reduce the infectivity of the influenza viruses indicates the prospects of researching these compounds as virucidal agents affecting extracellular virions. Among the studied triazolyl lupinine derivatives, compound **5** demonstrated the highest level of model virus (H1N1 and H3N2) inhibition. The obtained results of structure–activity relationships are quite important for the further design of new triazolyl lupinine derivatives, modified in the triazolyl ring. In the studied dose range of 0.03–1% (from 0.03 mg to 1 mg in 100 μL), none of the five lupinine derivatives achieved the LD_50_ value. Computer modeling results showed that the studied compounds have potential for use as neuraminidase inhibitors. The results of this research have not only revealed the capability of lupinine derivatives as antiviral agents for the first time, but also underscore the significance of rational drug design on the basis of plant quinolizine and quinolizidine alkaloids.

## Figures and Tables

**Table 1:** Virucidal activity of the studied samples **1**–**5**.

Compound 0.2%, 0.4 mg/ch.e. (8 mg/kg)	Decrease in Influenza Virus Infectivity Titer, lg	
	H3N2	H1N1
**1**	1.5	1.6
**2**	0.75	0.8
**3**	0.0	0.0
**4**	1.25	1.3
**5**	2.0	1.9

**Table 2:** Virus inhibitory activity of samples against influenza viruses.

Compound	Chemical Therapeutic Index of the Studied Sample	
	A/Almaty/8/98 (H3N2)	A/Vladivostok/2/09 (H1N1)
**1**	65.0	64.0
**2**	65.0	57.0
**3**	8.0	8.1
**4**	50.0	40.0
**5**	>100.0	>100.0
Tamiflu	10.3	11.0
Rimantadine	29.9	30

**Table 3:** The characteristics of the docking poses of compounds **1**–**5** in the HA and NA proteins.

Compound	Binding Energy, kcal/mol	Amino Acid Residues Within 3 Å from the Pose	Hydrogen Bonds
Hemagglutinin (PDB: 1RUY) *			
**1**	−7.1	Gly97H, Asp98H, Arg229H, Lys572I, Leu573I, Lys208J, Tyr209J, Asn210J, Arg211J	-
**2**	−7.2	Phe570I, Asn571I, Lys572I, Leu573I, Lys575I, Glu578I, Gln108J, Trp234J, Thr235J, Leu236J	Gln108J (NH_2_) with triazole nitrogen
**3**	−6.7	Asp98H, Asn571I, Lys572I, Leu573I, Ile100J, Asp101J, Lys208J, Tyr209J, Asn210J, Arg211J, Tyr233J, Trp234J	Tyr209J (OH) and Arg211J (NH_2_) with triazole nitrogen
**4**	−7.4	Asp98H, Phe99H, Asp101H, Tyr102H, Glu103H, Asn571I, Lys572I, Leu573I, Tyr209J	Asp101H (NH) with triazole nitrogen, Glu103H (COO^−^) weak with protonated triazole NH, Asn571I (NH_2_) weak with ether oxygen, Lys572I (NH^3+^) with aldehyde oxygen
**5**	−7.7	Asp98H, Phe99H, Asp101H, Tyr102H, Glu103H, Asn571I, Lys572I, Leu573I, Lys208J, Tyr209J, Asn210J, Arg211J	Asp101H (NH) with triazole nitrogen
Neuraminidase (PDB: 3BEQ)			
**1**	−8.3	Val116B, Ile117B, Arg118B, Leu134B, Thr135B, Gln136B, Gly147B, Thr148B, Val149B, Asp151B, Arg156B, Gln430B, Pro431B, Thr439B	Arg156B (NH_2_) with OCH_3_
**2**	−7.9	Val116B, Arg118B, Thr135B, Gln136B, Gly147B, Thr148B, Val149B, Asp151B, Arg156B, Gln430B, Pro431B, Thr439B	-
**3**	−7.5	Arg118B, Asp151B, Arg371B, Trp403B, Ile427B, Gln430B, Pro431B, Lys432B, Thr439B	Arg371B (NH_2_) with triazole nitrogen
**4**	−8.4	Val116A, Arg118A, Glu119A, Gln136A, Gly147A, Asp151A, Arg152A, Arg156A, Trp178A, Ser179A, Ile222A, Arg224A, Glu277A, Tyr406A, Trp438A, Thr439A	Arg156A (NH_2_) with triazole nitrogen, Thr439A (NH) with aldehyde oxygen
**5**	−9.6	Arg118B, Glu119B, Leu134B, Asp151B, Arg152B, Arg156B, Trp178B, Glu277B, Ser370B, Arg371B, Trp403B, Ser404B, Tyr406B, Ile427B, Pro431B, Lys432B	Arg118B and Arg371B with triazole nitrogen

* The docking poses in the HA cavity near the vestigial esterase and receptor binding subdomains (Site 1). The characteristics of the poses within the stem region of HA (Figure 5) are presented in Supplementary Table S1.

**Table 4:** Numbering and structural formulae of the studied compounds **1–5**.

Compound	IUPAC Name	Structural Formula
**1**	(1*S*,9a*R*)-1-{[4-(4-Methoxyphenyl)-1*H*-1,2,3-triazol-1-yl]methyl}octahydro-2*H*-quinolizine	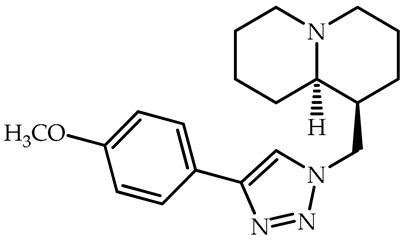
**2**	(1*S*,9a*R*)-1-{[4-(*m*-Tolyl)-1*H*-1,2,3-triazol-1-yl]methyl}octahydro-2*H*-quinolizine	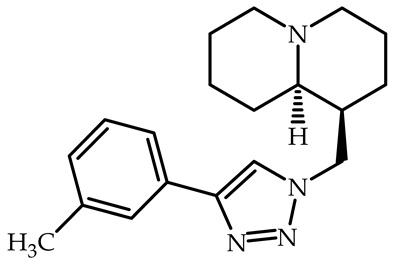
**3**	(1*S*,9a*R*)-1-[(4-Phenyl-1*H*-1,2,3-triazol-1-yl)methyl]octahydro-2*H*- quinolizine	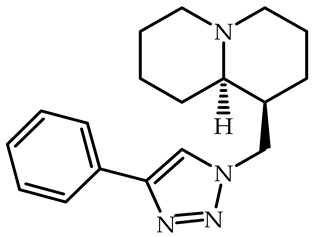
**4**	4-({1-[((1*S*,9a*R*)-Octahydro-2*H*-quinolizin-1-yl)methyl]-1*H*-1,2,3-triazol-4-yl}methoxy)benzaldehyde	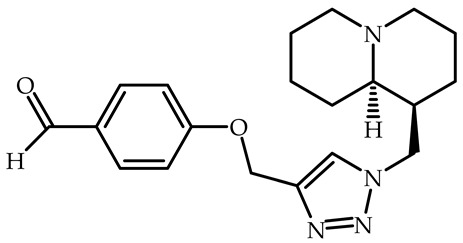
**5**	{1-[((1*S*,9a*R*)-Octahydro-2*H*-quinolizin-1-yl)methyl]-1*H*-1,2,3-triazol-4-yl}methyl-3-tert-butyl-2-hydroxy-5-ethylbenzoate	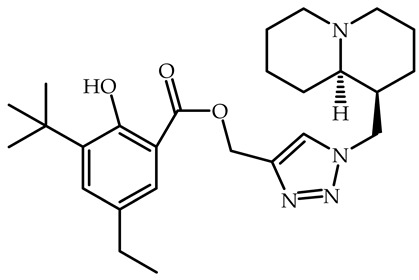

## Data Availability

Data are contained within the article.

## Supplementary Materials

The following supporting information can be downloaded at: https://www.mdpi.com/article/10.3390/molecules29235742/s1, Table S1: The characteristics of the best docking poses of compounds **1**–**5** in the stem region of hemagglutinin (PDB: 1RUY).
