# Supplementary material for: Antiviral Activity of (1S,9aR)-1-[(1,2,3-Triazol-1-yl)methyl]octahydro-1H-quinolizines from the Alkaloid Lupinine
Source: Molecules. 2024 Dec 5;29(23):5742. doi: 10.3390/molecules29235742 (PMC11644009; doi:10.3390/molecules29235742)
Supplement: Supplementary file 1 [file molecules-29-05742-s001.zip › molecules-3311680-supplementary.pdf]

Supplementary Materials

Antiviral activity of (1*S*,9a*R*)-1-[(1,2,3-triazol-1-yl)methyl]octahydro-1*H*-quinolizines from the alkaloid lupinine

Zhangeldy S. Nurmaganbetov [1,2], Oralgazy A. Nurkenov [1*], Andrei I. Khlebnikov[3], Serik D. Fazylov [1], Roza B. Seidakhmetova [4], Zhanar K. Tukhmetova [5], Altynaray T. Takibayeva [6], Gaukhar Khabdolda [5], Zhanar B. Rakhimberlinova [6], Aigul K. Kaldybayeva [7] and Elvira E. Shults [8*]

[1] Laboratory of Synthesis of Biologically Active Substances, Institute of Organic Synthesis and Coal Chemistry, Karaganda 100008, Kazakhstan; nzhangeldy@yandex.ru (Zh.S.N.); nurkenov_oral@mail.ru (O.A.N.); iosu8990@mail.ru (S.D.F.)
[2] School of Pharmacy, Karaganda Medical University, Karaganda 100008, Kazakhstan
[3] Kizhner Research Center, Tomsk Polytechnic University, Tomsk 634050, Russia; aikhl@chem.org.ru (A.I.Kh.)
[4] Department of Clinical Pharmacology and Evidence-Based Medicine, Karaganda Medical University, Karaganda, 100008, Kazakhstan; rozabat@mail.ru (R.B.S.)
[5] Department of Biomedicine, Karaganda Medical University, Karaganda, 100008, Kazakhstan; zhanar.tuhmetova@mail.ru (Zh.K.T.); khabdoldag@mail.ru (G.Kh.)
[6] Department of Chemistry and Chemical Technology, A. Saginov Karaganda Technical University, Karaganda 100027, Kazakhstan; altynarai81@mail.ru (A.T.T.); kargtu_tss@mail.ru (Zh.B.R.)
[7] Department of Pharmaceutical and Toxicological Chemistry, S.D. Asfendiyarov Kazakh National Medical University, Almaty 050012, Kazakhstan; aigul_240873@mail.ru (A.K.K.)
[8] N.N. Vorozhtsov Novosibirsk Institute of Organic Chemistry, Siberian Branch of the Russian Academy of Sciences, Novosibirsk 630090, Russia; schultz@nioch.nsc.ru (E.E.S.)
* Correspondence: nurkenov_oral@mail.ru (O.A.N.); schultz@nioch.nsc.ru (E.E.S.)

Table S1. The characteristics of the best docking poses of compounds **1-5** in the stem region of hemagglutinin (PDB: 1RUY).

Compound	Binding energy, kcal/mol	Amino acid residues within 3 Å from the pose	Hydrogen bonds
**1**	-8.3	Tyr594I, Asn595I, Leu598I, Leu602I, Glu603I, Arg606I, Asn595K, Leu598K, Leu599K, Leu602K, Glu603K, Arg606K, Tyr594M, Asn595M, Leu598M, Leu602M, Arg606M	-
**2**	-8.7	Tyr594I, Leu598I, Leu602I, Glu603I, Arg606I, Tyr594K, Asn595K, Leu598K, Leu599K, Leu602K, Glu603K, Arg606K, Asn595M, Leu598M, Leu602M, Arg606M	-
**3**	-8.2	Tyr594I, Leu598I, Leu602I, Glu603I, Arg606I, Asn595K, Leu598K, Leu599K, Leu602K, Glu603K, Arg606K, Leu598M, Leu602M, Arg606M	-
**4**	-8.4	Tyr522I, Ser540I, Asn543I, Phe610I, Asn614I, Asn617I, Leu618I, Lys621I, Thr22L, Leu24L, Glu25L, Arg322L, Gly501M, Gly508M	Ser540I (OH) weak with triazole nitrogen, Arg322L (NH_2) with the aldehyde oxygen
**5**	-9.2	Gly504K, Gly508K, Phe509K, Tyr619K, Ser540M, Asn617M, Leu618M, Glu620M, Lys621M, Ser624M	Ser624M (OH) with the ester carbonyl oxygen and *ortho*-OH hydrogen
